# A case report of pelviscopic resection of lipoleiomyoma originating from the uterine cervix in a postmenopausal woman

**DOI:** 10.1097/MD.0000000000030665

**Published:** 2022-09-30

**Authors:** Yun Sook Kim, Ji Hye Lee

**Affiliations:** a Department of Obstetrics and Gynecology, Soonchunhyang Cheonan University Hospital, Cheonan, Korea; b Pathology, Soonchunhyang University College of Medicine, Soonchunhyang University Cheonan Hospital, Cheonan, Korea.

**Keywords:** cervix, lipoleiomyoma, pelviscopy, postmenopause

## Abstract

Lipoleiomyoma is a rare neoplasm of the uterus. It is considered a variant of uterine myomas. Its reported incidence varies from 0.03% to 0.2%. Lipoleiomyoma consists of variable proportions of mature lipocytes and smooth muscle cells. These tumors generally occur in asymptomatic obese perimenopausal or postmenopausal women. About 90.7% of lipoleiomyomas arise from the uterine corpus, with only 6.5% arising from the cervix. When it occurs in the cervix, it is difficult to diagnose and treat it. We report an uncommon case of pelviscopic resection of uterine cervical lipoleiomyoma showing continuous growth after menopause.

**Patient concerns:**

A 55-year-old postmenopausal woman was diagnosed with 40 mm-sized uterine myoma 4 years ago. The size of the mass increased to 58 mm in the last year.

**Diagnoses:**

An ultrasound scan revealed a 58 × 34-mm-sized round hyperechogenic and barely vascularity mass that appeared to have originated on the left side of the uterine cervix. Final pathologic findings showed lipoleiomyoma.

**Interventions:**

After admission to the hospital, we performed pelviscopic removal of uterine lipoleiomyoma and both tubes. Microscopic examination revealed a significant amount of fat cells between muscle cells.

**Outcomes:**

Surgeries were successful. The patient had been followed up regularly for three years after surgery. She did not experience any complications. She remained disease-free.

**Lessons:**

Although lipoleiomyomas mainly occur in postmenopausal women, they can also occur in the uterine cervix. They can increase in size after menopause. They can be removed laparoscopically. If a hyperechoic mass occurred in the uterus after menopause that keeps growing without symptoms, a differential diagnosis of lipoleiomyomas must be performed.

## 1. Introduction

“Myolipoma of soft tissue” was thought to be first described in 1991 by Meis and Enzinger.^[1]^ Lipomatous uterine tumors are unusual benign neoplasms.^[2]^ Most cases of lipoleiomyoma cannot be distinguished clinically from leiomyoma. They can be diagnosed typically by gross glistening yellow appearance of the tumor. Most patients are asymptomatic. Occasionally they may complain of vaginal bleeding or pelvic pain.^[3]^ Lipoleiomyomas are predominantly located in the uterus, although, extrauterine locations have also been reported.^[4]^ Their ultrasonographic findings include a well-defined, homogeneously echogenic lesion without vascularity.^[5]^ The tumor consists of long intersecting bundles of bland and, smooth muscle cells admixed with mature fat cells.^[6]^ Surgical treatment is suggested for symptomatic lesions or when a diagnosis is in doubt. Here we report a very rare case of lipoleiomyoma of the uterine cervix with a literature review. It was removed pelviscopically. This case report can be helpful for clinicians.

## 2. Case presentation

**Figure 1.**
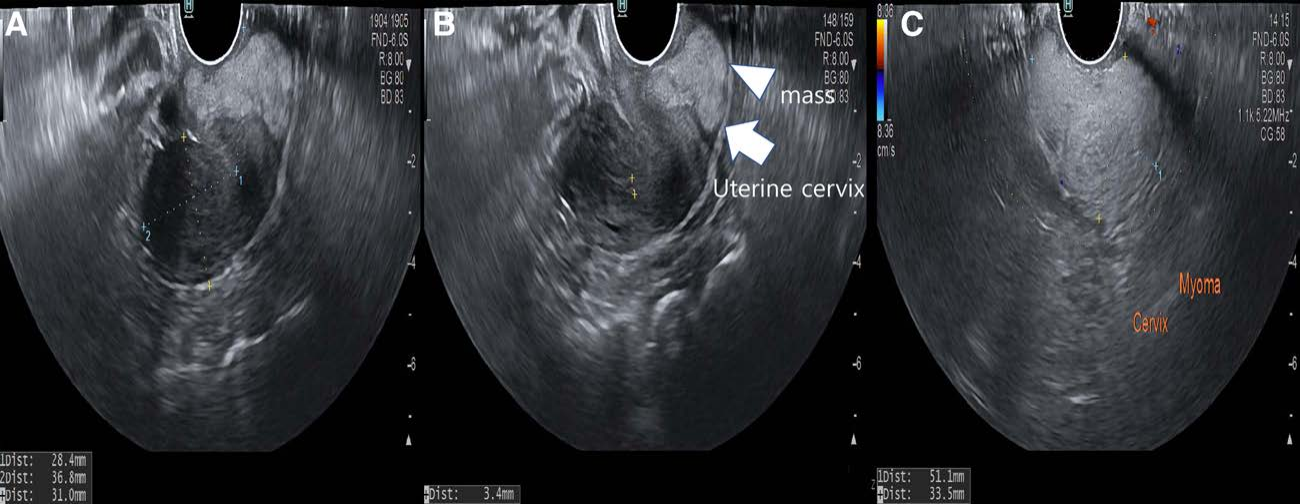
Transvaginal ultrasound findings. (A, B) A well-defined, hyperechogenic mass measuring 58 34 mm ×in size was seen in the cervix. Thickness of the endometrium was 3 mm. Both ovaries and tubes were normal on transvaginal ultrasound. (C) There was no vascularity in the mass on color Doppler.

**Figure 2.**
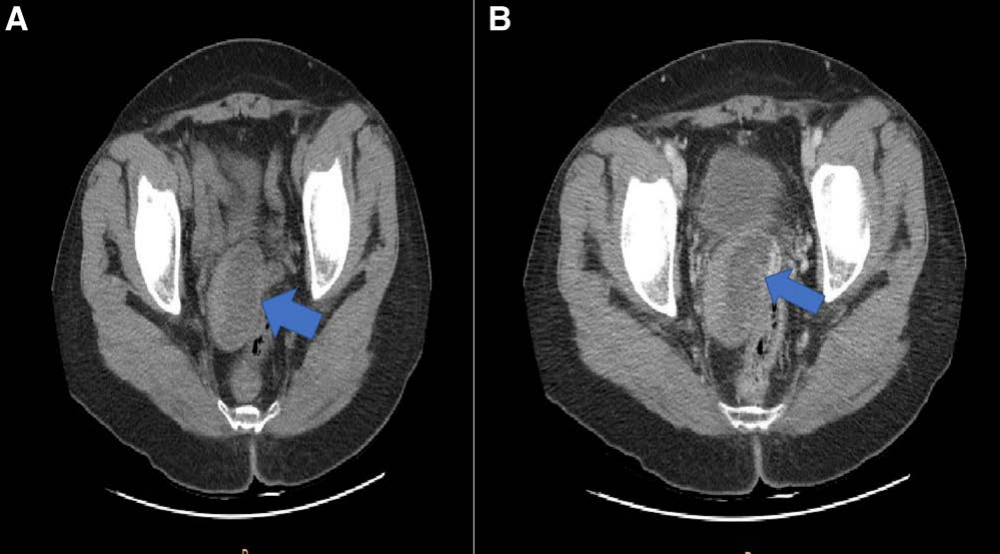
APCT findings. (A, B) Low attenuated benign looking lesions (arrow) arising from the uterine cervix were seen well-marginated, corresponding to fat density. There were no cystic changes or calcification within the lesion.

**Figure 3.**
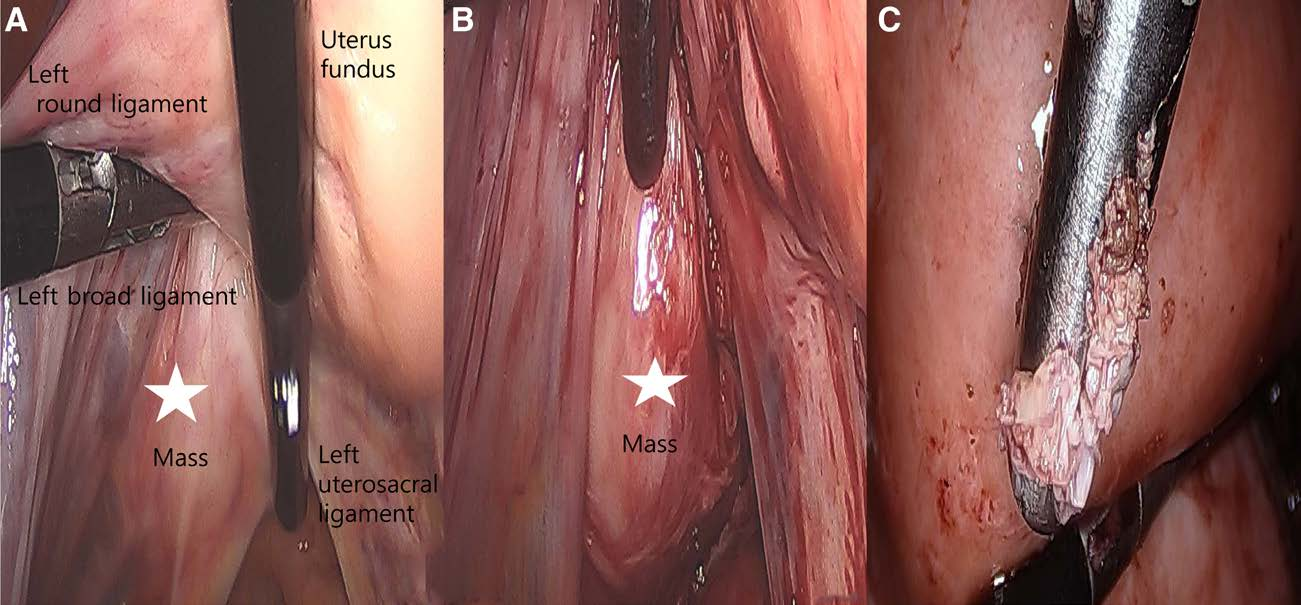
Laparoscopic findings. (A, B) The mass was round, elongated, and originated from the left side of the uterine cervix. The mass (asterisk) was located below the left uterosacral ligament and broad ligament. (C) Specimen was different from a typical appearance of uterine leiomyoma by being pale whitish, and having a somewhat dry and softer consistency on its several fragments.

**Figure 4.**
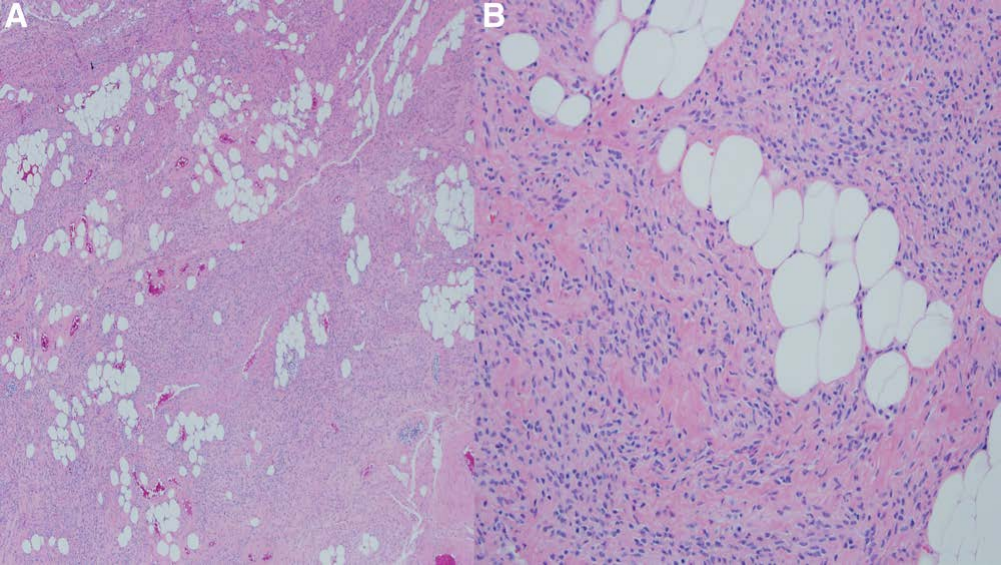
Microscopic findings. (A) Histological examination of the biggest nodule showed a mixture of bland, spindle-shaped smooth muscle cells without nuclear atypia in a whorled pattern with admixed mature adipocytes (H&E, ×40). (B) Nuclei of smooth muscles were elongated with finely dispersed chromatin and small nucleoli. Between these muscle cells, significant amounts of fat cells were visible. The adipose component was entirely mature without any lipoblasts (H&E, ×400).

A 55-year-old Asian woman, gravida 2, para 2, presented with cervical mass that were increasing in size. She had no medical or surgical history. She was 163 cm tall with a weight of 62 kg at the time of her visit. She went through menopause 5 years ago. She had not taken any hormonal replacement therapy. She was found to have a 40 mm-sized mass on the cervix in an examination 4 years ago. However, the size suddenly increased to 58 mm last year. She had no symptoms. After menopause, the size of the mass increased, making it necessary to make differential diagnosis from cancer. She was transferred to our hospital. Her Pap (Papanicolaou) smear done 6 months ago was normal. Her cancer antigen (CA) 125 (0–35 U/mL) level was 10.5 U/mL. All other laboratory values including lipid profiles were within normal ranges. Gynecologic examination revealed no abnormalities of the vulva or, cylindrical vaginal portion of the cervix. No evident pathological change was detectable. A well-defined, hyperechogenic mass measuring 58 34 mm was seen in the cervix. Thickness of the endometrium was 3 mm. Both ovaries and tubes were normal on transvaginal ultrasound. There was no vascularity in the mass on color Doppler (Fig.1A–C). In abdomen and pelvis computed tomography (APCT), low attenuated benign-looking lesions arising from the uterine cervix were seen well-marginated, corresponding to fat density. There were no cystic changes or calcification within the lesion (Fig. 2A,B). The patient strongly desired to preserve the uterus. Thus, we underwent pelviscopic resection of mass with bilateral salpingectomy (Fig. 3A,B). On gross examination of the specimen, the mass was measured to be a total of 6.8 × 4.5 × 0.6 cm in size with many fragments. The specimen differed from a typical appearance of uterine leiomyoma by being pale whitish and having a somewhat dry and softer consistency on its several fragments (Fig. 3C). Her fallopian tubes appeared grossly normal. Histological examination of the biggest nodule showed a mixture of bland, spindle-shaped smooth muscle cells without nuclear atypia in a whorled pattern with admixed mature adipocytes. The nuclei of smooth muscles were elongated with finely dispersed chromatin and small nucleoli. Between these muscle cells, significant amounts of fat cells were visible. The adipose component was entirely mature without any lipoblasts in hematoxylin and eosin (H&E) staining (Fig. 4A,B). Based on the above findings, the tumor was diagnosed as a benign lipoleiomyoma. Sections from both tubes showed unremarkable histologically. The patient has been followed-up regularly. She remains disease-free for 3 years after the surgery.

## 3. Discussions

Uterine lipoleiomyomas resulting from degeneration of smooth muscle cells in an ordinary leiomyoma are rare benign tumors of the uterus. In 2014, Akbulut et al reported that patients with lipoleomyomas ranged in age from 34 years to 77 years (mean: 55.49 years) with sizes ranging from 0.5 cm to 55 cm in diameter (mean: 5.50 cm). Sixty-nine (90.7%) tumors were in the uterine corpus while only 5 (6.5%) were in the cervix.^[7]^ The pathogenesis of lipoleiomyomas remains obscure. Estrogen deficiency as occurs in peri- or post-menopausal period might promote abnormal intracellular storage of lipids. Estrogen deficiency may not only induce proliferation of the lipomatous component in lipoleiomyomas, but also be associated with many endocrine such as obesity, hyperlipidemia, and hypertension. Therefore, uterine lipoleiomyomas are characterized by progressive enlargement even after menopause.^[8]^ She had no symptoms, although the mass increased in size after menopause, making it necessary to make a differential diagnosis from cancer. The differential diagnosis of uterine lipoleiomyoma includes ovarian teratoma, benign pelvic or uterine lipoma, liposarcoma, extra-adrenal myelolipoma, angiomyolipoma, lipoblastic lymphadenopathy, and retroperitoneal cystic hamartomas.^[9]^ Although most patients are asymptomatic, they can present with symptoms similar to leiomyomas of the same size and location. Lipoleiomyomas may induce complications through compression caused by the mass, although it has low malignant potential. Symptoms include, but are not limited to, abdominal and pelvic pain, palpable mass, and urinary symptoms like incontinence, urgency, and frequency.^[10]^ Ultrasonographic findings are hyperechoic with a partially hypoechoic rim. The rim likely represents a layer of myometrium surrounding the fatty central component with posterior acoustic attenuation and often poor or no vascularity on color Doppler examination.^[11]^ APCT predominantly reveals fat-containing mass arising from the uterus. It is seen as well-marginated, often containing areas of soft tissue density. Magnetic resonance imaging (MRI) shows secondary to the predominant fatty component in the lesion. Hyperintensity is seen on T1 weighted sequences. Hypointense on T2 fat suppression sequences is also noted. Additionally, fat suppression techniques can be used to verify the diagnosis as most lesions show fat suppression.^[12]^ We did not perform additional MRI as the mass was benign on APCT. Lipoleiomyomas when small and asymptomatic usually do not require treatment. They are clinically similar to leiomyomas. Treatment depends on clinical symptoms and the size/location of the lesion. Uterine artery embolization or surgical excision can be performed, as indicated.^[13]^ For uterine fibroids, regular follow-up for changes in size or symptoms has been suggested. However, the rate of growth is higher for lipoleiomyomas than for conventional uterine myomas, the latter of which tend to decrease in size after menopause. It has been reported that the relatively rapid growth rate of lipoleiomyomas mimics that of malignant tumors. For symptomatic lesions or when a diagnosis is in doubt, surgical treatment is recommended. General surgical options for lipoleiomyomas include myomectomy through abdominal, laparoscopy or hysteroscopy, and hysterectomy with or without bilateral salpingo-oophorectomy through the abdomen or vagina.^[14]^ Histological examinations of lipoleiomyomas show a mixture of bland, spindle-shaped smooth muscle cells without nuclear atypia in a whorled pattern with admixed mature adipocytes. A significant amount of fat cells between muscle cells can be seen. The adipose component is entirely mature without any lipoblasts.^[15]^

## 4. Conclusions

Although the patient’s history and imaging studies play an important role in preoperative diagnosis and site of the lipoleiomyoma, it is the pathological examination that can confirm the diagnosis finally. In the present case, the patient underwent laparoscopic lipoleiomyoma resection. She had no recurrence at 3 years of follow-up. This study is of great value in that few studies have reported lipoleiomyomas removed by laparoscopy so far.

## Acknowledgments

The authors are grateful to Soonchunhyang University Cheonan Hospital for their assistance and encouragement.

## Author contributions

Conceptualization, data curation, investigation, and writing of original draft preparation: Yun Sook Kim. Writing-review, Pathology: Ji Hye Lee. All authors read and approved the final manuscript.

## References

[1] Oh MH Cho IC Kang YI. A case of retroperitoneal lipoleiomyoma. J Korean Med Sci. 2001;16:250–2. 11306758 10.3346/jkms.2001.16.2.250 PMC3054726

[2] Oh SR Cho YJ Han M Bae JW Park JW Rha SH. Uterine lipoleiomyoma in peri or postmenopausal women. J Menopausal Med. 2015;21:165–70. 26793683 10.6118/jmm.2015.21.3.165 PMC4719092

[3] Rampersad F Verma S Diljohn J Persad V Persad P. Uterine lipoleiomyoma presenting with pelvic pain in a post-menopausal woman. Cureus. 2021;13:e14929. 34123628 10.7759/cureus.14929 PMC8189540

[4] Schaefer SL Strong AL Bahroloomi S. Large intraperitoneal lipoleiomyoma in a premenopausal woman: a case report. World J Surg Oncol. 2021;19:144. 33964957 10.1186/s12957-021-02256-9 PMC8106865

[5] Maebayashi T Imai K Takekawa Y. Radiologic features of uterine lipoleiomyoma. J Comput Assist Tomogr. 2003;27:162–5. 12703006 10.1097/00004728-200303000-00010

[6] Oh SR Cho YJ Han M Bae JW Park JW Rha SH. Uterine lipoleiomyoma in peri or postmenopausal women. Korean J Clin Oncol. 2015;21(3):165–70. 10.6118/jmm.2015.21.3.165 PMC4719092 26793683

[7] Akbulut M Gündoğan M Aygün Yörükoğlu A. Clinical and pathological features of lipoleiomyoma of the uterine corpus: a review of 76 cases. Balkan Med J. 2014;31:224–9. 25625021 10.5152/balkanmedj.2014.13079 PMC4299967

[8] Yuan Y Che L Zhao T Yu M. Pathogenesis, diagnosis and treatment of uterine lipoleiomyoma: a review. Biomed Pharmacother. 2021;142:1120113. 10.1016/j.biopha.2021.112013 34388526

[9] Kim JC. Exophytic lipoleiomyoma of the uterus mimicking ovarian teratoma: a case report. J Korean Radiol Soc. 2006;55:285–8.

[10]] LeeTHBaekSJ. Extraabdominal parasitic lipoleiomyoma. Korean J Clin Oncol. 2021;17:48–51.10.14216/kjco.21008PMC994274536945208

[11] Lee SJ Chae HD Jeong BD. A clinical review of uterine lipoleiomyoma: a study for value and limitations of radiologic evaluation in preoperative diagnosis of lipoleiomyoma. Korean J Obstet Gynecol. 2012;55:953–7.

[12] Nazir HM Mehta S Seena CR Kulasekaran N. Uterine lipoleiomyoma: a report of two cases. J Clin Imaging Sci. 2017;7:26. 28717557 10.4103/jcis.JCIS_13_17 PMC5508403

[13] Daga S Phatak S Chaudhari K. Lipoleiomyoma-A rare genign neoplasm. J Datta Maghe Inst Med Sci Univ. 2019;14:254–5.

[14] El-agwany AS. Lipoleiomyoma of the uterine cervix: an unusual variant of uterine leiomyoma. Egypt J Radiol Nucl Med. 2015;46:211–3.

[15] Wang X Kumar D Seidman JD. Uterine lipoleiomyomas: a clinicopathologic study of 50 cases. Int J Gynecol Pathol. 2006;25:239–42. 16810060 10.1097/01.pgp.0000192273.66931.29

